# Nrf2 activation through the PI3K/GSK-3 axis protects neuronal cells from Aβ-mediated oxidative and metabolic damage

**DOI:** 10.1186/s13195-019-0578-9

**Published:** 2020-01-13

**Authors:** Krystal Sotolongo, Jorge Ghiso, Agueda Rostagno

**Affiliations:** 1 Department of Pathology, New York University School of Medicine, 550 First Avenue, New York, NY 10016 USA; 2 Department of Psychiatry, New York University School of Medicine, 550 First Avenue, New York, NY 10016 USA

**Keywords:** Alzheimer’s disease, Amyloid-β, Mitochondria, Methazolamide, Melatonin, Trolox, Oxidative stress, Cell metabolism and bioenergetics, Oxygen consumption, Cellular respiration

## Abstract

**Background:**

Mounting evidence points to a crucial role of amyloid-β (Aβ) in the pathophysiology of Alzheimer’s disease (AD), a disorder in which brain glucose hypometabolism, downregulation of central elements of phosphorylation pathways, reduced ATP levels, and enhanced oxidative damage coexist, and sometimes precede, synaptic alterations and clinical manifestations. Since the brain has limited energy storage capacity, mitochondria play essential roles in maintaining the high levels of energy demand, but, as major consumers of oxygen, these organelles are also the most important generators of reactive oxygen species (ROS). Thus, it is not surprising that mitochondrial dysfunction is tightly linked to synaptic loss and AD pathophysiology. In spite of their relevance, the mechanistic links among ROS homeostasis, metabolic alterations, and cell bioenergetics, particularly in relation to Aβ, still remain elusive.

**Methods:**

We have used classic biochemical and immunocytochemical approaches together with the evaluation of real-time changes in global energy metabolism in a Seahorse Metabolic Analyzer to provide insights into the detrimental role of oligAβ in SH-SY5Y and primary neurons testing their pharmacologic protection by small molecules.

**Results:**

Our findings indicate that oligomeric Aβ induces a dramatic increase in ROS production and severely affects neuronal metabolism and bioenergetics. Assessment of global energy metabolism in real time demonstrated Aβ-mediated reduction in oxygen consumption affecting basal and maximal respiration and causing decreased ATP production. Pharmacologic targeting of Aβ-challenged neurons with a set of small molecules of known antioxidant and cytoprotective activity prevented the metabolic/bioenergetic changes induced by the peptide, fully restoring mitochondrial function while inducing an antioxidant response that counterbalanced the ROS production. Search for a mechanistic link among the protective small molecules tested identified the transcription factor Nrf2—compromised by age and downregulated in AD and transgenic models—as their main target and the PI3K/GSK-3 axis as the central pathway through which the compounds elicit their Aβ protective action.

**Conclusions:**

Our study provides insights into the complex molecular mechanisms triggered by oligAβ which profoundly affect mitochondrial performance and argues for the inclusion of small molecules targeting the PI3K/GSK-3 axis and Nrf2-mediated pathways as part of the current or future combinatorial therapies.

## Background

Alzheimer’s disease (AD), the most common type of dementia, is neuropathologically characterized by the presence of hyperphosphorylated tau in intraneuronal neurofibrillary tangles and the deposition of amyloid-β (Aβ) in the brain parenchyma and cerebral vasculature [1]. Although it remains unclear what primarily triggers and drives the progression of AD, strong evidence supports a pathogenic role for Aβ oligomeric conformations [2, 3]. It is now considered that the transition from soluble monomeric species normally present in body fluids to the oligomeric, protofibrillar, and end-point fibrillar assemblies contributes significantly to disease pathogenesis. Intermediate oligomeric and protofibrillar forms seem to display the most potent effects in neuronal cells inducing synaptic disruption, neurotoxicity, and ultimately neurodegenerative cell death [3, 4].

The molecular mechanisms leading to AD pathophysiology are complex and not fully elucidated with mounting evidence highlighting a central role for mitochondrial dysfunction taking place at the early stages of the disease and supporting a causative role for these abnormalities in AD pathogenesis [5, 6]. Previous studies from our lab as well as the work of others indicate that Aβ accumulation leads to a cascade of events affecting mitochondrial function not only in neurons and glial cell populations but also in cells of the cerebral microvasculature [7–13]. In this sense, our work has described in detail the Aβ-elicited initiation of apoptotic pathways demonstrating induction of caspase-mediated mitochondrial pathways with changes in mitochondrial membrane potential, Bax (Bcl-2-associated X protein) translocation, and cytochrome c release to the cytosol, events that ultimately lead to cellular death [7–9, 14].

Mitochondria are increasingly recognized as subcellular organelles essential for generating the energy that fuels cell function while simultaneously monitoring cellular health and acting as regulators of programmed cell death. Under physiological conditions, the brain requires high metabolic energy to sustain transport systems at endothelial barriers and maintain ion gradients across membranes critical for the generation of action potentials. Based on the limited glycolytic capacity of neurons, these cells are highly dependent on mitochondria-mediated aerobic oxidative phosphorylation (OXPHOS) for their energetic needs. As the main consumers of oxygen, mitochondria are also the main generators of toxic reactive oxygen species (ROS) as products of normal cellular respiration [5, 15]. Under circumstances in which these radicals overwhelm the neuronal capacity to neutralize them, irreversible damage to cellular components occurs resulting in oxidative damage to nucleic acids, proteins, and lipids, neuronal injury, initiation of apoptotic cascades facilitating the formation of the apoptosome, and subsequent cell death [16].

The pathogenic relevance of mitochondrial function for AD has boosted interest in pharmacologic targeting of these organelles which is currently actively pursued as potential therapeutic strategies. Along this line, different antioxidant agents have been tested to counterbalance oxidative stress generation in in vitro paradigms, in different animal models of various neurodegenerative disorders, and for some of them in humans. Among the many molecules tested are the saffron-derived compound crocin, polyphenols like resveratrol, and vitamin E [17–21]. More recently, two specific inhibitors of cytochrome c release lacking additional multifunctional activity—methazolamide (MTZ) and melatonin (MEL)—were identified by screening a library of compounds (NINDS Drug Screening Consortium) on isolated mitochondria [22] and proved to be neuroprotective in models of Huntington’s disease and ischemic injury [22, 23]. Work from our lab has shown that MTZ rescues microvascular, glial, and neuronal cells from Aβ-mediated apoptosis, restoring mitochondrial membrane potential, preventing cytochrome c release to the cytoplasma, precluding activation of mitochondria-associated caspase-9, and ultimately inhibiting the activation of terminal caspases and the induction of cell death mechanisms [7, 8, 24]. Additional research has also shown a protective role of MEL from Aβ-mediated mitochondrial alterations through the compound’s antioxidant features [25, 26]. This protective effect extends beyond MEL radical scavenging properties preserving mitochondrial membrane potential and exerting broad effects on mitochondrial activity attenuating activation of initiator caspase-9 and the effector caspase-3/7 [22, 27–29].

The work presented herein expands current knowledge on the cellular pathways detrimentally affected by Aβ providing an insight into the mechanisms by which MTZ, MEL, and the vitamin E analog Trolox prevent not only the formation of oxidative radicals but also the concomitant metabolic/bioenergetic neuronal alterations. The data identify the ability of the three small molecule compounds to activate Nrf2, a key central regulator of the antioxidant response, as a common mechanistic link responsible for their protective activity. Through a combination of real-time metabolic/bioenergetic assessments and immunocytochemical approaches together with ELISA and dot and Western blot analyses of nuclear extracts, as well as the use of specific kinase inhibitors, the manuscript clearly demonstrates that MTZ, MEL, and Trolox protective effect on Aβ-mediated alterations relies on the activation of the transcription factor Nrf2 through the PI3K/Akt axis. The work unveils new targets for potential pharmacologic interventions opening new routes of translational research for the future development of novel therapeutic applications.

## Material and methods

### Materials

Methazolamide (MTZ, *N*-[5-(aminosulfonyl)-3-methyl-1,3,4-thiadiazol-2(3H)-ylidene]-acetamide), melatonin (MEL, *N*-acetyl-5-methoxy tryptamine), Trolox (6-hydroxy-2,5,7,8-tetramethylchroman-2-carboxylic acid), and sulforaphane (SFN, 1-isothiocyanato-4-(methylsulfinyl)-butane) were procured from Sigma. The PI3K inhibitors Wortmannin and LY294002 were from Millipore-Sigma (Burlington, MA) and Cell Signaling (Danvers, MA), respectively. The GSK-3 inhibitor SB216763 was purchased from Cell Signaling.

### Synthetic peptides

Synthetic homologs of Aβ1–42 were synthesized using *N-tert-*butyloxycarbonyl chemistry at ERI Amyloid Laboratory (Oxford, CT). Peptides were purified by reverse-phase high-performance liquid chromatography on a Vydac C4 column (Western Analytical, Murrieta, CA), molecular masses were corroborated by matrix-assisted laser desorption ionization time-of-flight (MALDI-TOF) mass spectrometry, and concentrations were assessed by amino acid analysis, as previously reported [8]. Peptides were dissolved at a concentration of 1 mg/ml in 1,1,1,3,3,3, hexafluoro-isopropanol (HFIP; Sigma Chemical Co., St. Louis, MO) and incubated overnight a room temperature (RT), a pretreatment that breaks down β-sheet structures and disrupts hydrophobic forces leading to monodisperse amyloid subunit preparations [30]. Following lyophilization, peptides were thoroughly dissolved to 10 mM in dimethyl sulfoxide (DMSO, Sigma), brought up to 1 mM with deionized water, and further diluted in culture media to the desired concentrations indicated below, in agreement with our previously reported protocols [8, 31].

### Cell cultures

Immortalized SH-SY5Y neuroblastoma cells were obtained from the American Type Culture Collection (ATCC, Manassas, VA, USA) and maintained in DMEM/F12 medium (Mediatech, Manassas, VA) with 10% fetal bovine serum and 1% penicillin/streptomycin using standard protocols from our laboratory [31]. Primary cortical neuron cultures were prepared from embryonic day 18 rat brain tissue (Sprague Dawley; Charles River, Sharon, MA), in compliance with the New York University School of Medicine Institutional Animal Care and Use Committee, as described [32]. After dissection, the cortices were incubated 15 min at 37 °C in PBS/glucose/HEPES buffer (33 mM glucose/10 mM HEPES in 10 mM phosphate-buffered saline containing 138 mM NaCl and 2.7 mM KCl, pH 7.4) added of 0.25% trypsin, followed by mechanical disruption. Cells were resuspended in DMEM, seeded on poly-d-lysine-coated plates or glass coverslips depending on the subsequent experimental use; after 1 h, the media were changed to Neurobasal (Thermo Fisher Scientific/Invitrogen, Carlsbad, CA) containing 2% B27, glutamine, and penicillin/streptomycin. A one-time dose of the antimitotic drug AraC (1 μM, Sigma Chemical Co., St. Louis, MO) was added after 6 days in culture to inhibit growth of dividing cells [32]. Purity of the resulting cultures was assessed by immunofluorescence (IF) evaluation of the neuronal marker neurofilament protein and astroglial contamination estimated by assessment of glial fibrillary acidic protein (GFAP), as described below. Under these experimental conditions, primary cultures consisted of > 90% neurons.

### Assessment of Aβ oligomerization

#### Dot blot analysis

The presence of oligomeric forms of Aβ42 in the culture supernatants from Aβ-challenged SH-SY5Y cells treated with Aβ42 (10 μM; 24 h) in the presence/absence of MTZ (300 μM), MEL (100 μM), or Trolox (300 μM) was assessed by dot blot using rabbit polyclonal A11 anti-oligomer antibody (Thermo Fisher Scientific/Invitrogen) [33], as we previously described [8, 34]. Briefly, 80 μl of each of the culture supernatants was loaded onto a nitrocellulose membrane assembled into a Bio-Dot Microfiltration Apparatus (Bio-Rad, Hercules, CA). As a negative control for Aβ oligomer formation, the membranes were loaded with freshly solubilized HFIP-treated synthetic Aβ42 (800 ng). In all cases, samples were allowed to diffuse passively for 30 min before vacuum application and the membrane was subsequently blocked in situ for 1 h with 1% nonfat milk in TBST, followed by vacuum application and two washes with TBST. After removal from the dot blot apparatus and further blocking with 5% milk in TBST [1 h, room temperature (RT)], the membrane was incubated overnight with A11 antibody (1:1000) followed by HRP-conjugated anti-rabbit secondary antibody (GE Healthcare Life Sciences, Marlborough, MA; 1:2000, 1 h at RT). As a loading control, a set of wells filled with either the respective cell supernatants or the freshly solubilized synthetic Aβ42 was incubated with 4G8 monoclonal anti-Aβ antibody (BioLegend, San Diego, CA; 1:1000, overnight at 4 °C) followed by HRP-conjugated anti-mouse IgG (GE Healthcare Life Sciences; 1:2000, 1 h at RT). In all cases, immunoreactivity was evaluated by enhanced chemiluminescence (ECL, SuperSignal West Dura Extended Duration Substrate; Thermo Fisher Scientific/Pierce, Waltham, MA) and densitometric quantification of signal intensities assessed with ImageJ software (https://imagej.nih.gov).

#### Electron microscopy

The presence of oligomeric forms of Aβ42 in the culture supernatants was additionally assessed by electron microscopy using our previously described protocols [8, 14]. Briefly, 3 μl aliquots were placed onto carbon-coated 400-mesh Cu/Rh grids (Ted Pella, Inc., Redding, CA, USA) and stained with 1% uranyl acetate in distilled water (Polysciences, Inc., Warrington, PA, USA). Stained grids were examined in a Philips CM-12 transmission electron microscope and photographed with a Gatan (4 k × 4 k) digital camera at the NYU School of Medicine Microscopy Laboratory Core Facility.

### Detection of reactive oxygen species

Generation of ROS was evaluated by IF using CellROX deep red (Thermo Fisher Scientific/Invitrogen)—a probe recognizing different ROS species including peroxyl/hydroxyl radicals and peroxynitrite—using previously reported lab methodologies [35, 36]. Primary cortical neurons and SH-SY5Y cells were seeded on poly-d-lysine-coated glass coverslips at a density of 300,000 cells/coverslip, and challenged 24 h with increasing concentrations of Aβ1–42 (0–1 μM for primary neurons, 0–25 μM in the case of SH-SY5Y), in the presence or absence of MTZ (300 μM), MEL (100 μM), or Trolox (300 μM). After peptide treatment, cells were incubated with CellROX (5 μM, 30 min at 37 °C) and Hoechst stain (1 μg/ml; Immunochemistry Technologies, Bloomington, MN) followed by fixation in 4% paraformaldehyde (PFA). Images were acquired in a Nikon Eclipse Ti microscope and analyzed using ImageJ.

### Neuronal bioenergetic function and rate of oxidative metabolism of glucose

Global neuronal cell metabolic/bioenergetic profiles were evaluated with XF Cell Mito Stress Assay in a Seahorse XFe24 analyzer (Seahorse Bioscience/Agilent Technologies, North Billerica, MA)—available through the NYU School of Medicine Translational Research Core—which allows real-time metabolic analysis in live cells. Primary neurons and SH-SY5Y cells were plated on PDL-coated Seahorse Xe24 plates (30,000 and 80,000 cells/per well, respectively) and challenged 24 h with various concentrations of Aβ1–42 (0–1 μM for primary neurons and 0–20 μM for neuroblastoma SH-SY5Y) in the presence or absence of 300 μM MTZ, 100 μM MEL, or 300 μM Trolox. Cells were subsequently washed with XF assay media or artificial CSF [120 mM NaCl, 3.5 mM KCl, 1.3 mM CaCl_2_, 0.4 mM KH_2_PO_4_, 1 mM MgCl_2_, 5 mM HEPES, 5.0 mM glucose, 0.4% bovine serum albumin (BSA), pH 7.4] in the case of SHSY5Y cells or primary neurons, respectively. After washing, cells were further incubated in the respective media (37 °C, 1 h) in a CO_2_-free incubator to further purge CO_2_ and allow temperature/pH equilibration before each set of measurements in the metabolic analyzer. Each plate contained four wells not seeded with neurons to serve as blank controls for temperature-sensitive fluctuations in O_2_ fluorophore emission [37]. Following measurements of resting respiration, cells were sequentially treated with oligomycin (1 μM) for the assessment of non-phosphorylating oxygen consumption rate (OCR), with the mitochondrial uncoupler FCCP (2 μM) for the evaluation of maximal OCR, and with a combination of antimycin A and rotenone (both at 0.5 μM) for estimation of extra-mitochondrial OCR. In all cases, three measurements were recorded, each one over a 2-min interval followed by 2-min mixing and 2-min incubation.

At the end of the Seahorse runs, to allow a comparison among different experiments, data were normalized to the total cell amount per well as estimated by Janus Green Whole-Cell Stain (Sigma), an assay developed for anchorage-dependent cell cultures [38]. Briefly, cells were immediately fixed in 4% PFA and incubated with Janus Green (0.2% ethanol solution, 5 min). After removal of excess dye by ultrapure water washes, bound dye was eluted by 10 min incubation with 0.5 M HCl (0.1 ml/well) and evaluated at 595 nm in a microplate reader (Packard SpectraCount, Cole-Parmer, Vernon Hills, IL). In all cases, the metabolic parameters of the assay—basal and maximal respiration, proton leak, and ATP production through oxidative phosphorylation—were calculated with Agilent/Seahorse XF Report Generator software and expressed as OCR in pmol/min. The results are illustrated as means ± standard error from at least three to six independent experiments performed in triplicate.

### Assessment of Nrf2 pathway activation

Evaluation of Nrf2 activation by MTZ, MEL, and Trolox in the presence or absence of Aβ was performed through assessment of the translocation of the transcription factor to the nuclei by immunocytochemistry and dot and Western blot analysis of nuclear fractions, as well by ELISA evaluation of Nrf2 activity in these nuclear extracts.

#### Immunocytochemistry

SH-SY5Y cells and rat primary neurons were plated on PDL-coated coverslips and challenged with MTZ, MEL, or Trolox in the presence of varying Aβ42 concentrations, as described above. After treatment, cells were fixed with 4% PFA, blocked with PBS containing 20 mg/ml BSA/0.3% Triton X-100, and incubated with rabbit polyclonal anti-Nrf2 antibody (Thermo Fisher/Invitrogen; 1:500 in PBS containing 5 mg/ml BSA, 2 h at RT). After the primary antibody incubation, cells were subsequently reacted with Alexa Fluor 488-conjugated anti-rabbit IgG (Thermo Fisher/Invitrogen; 1:200 in PBS with 5 mg/ml BSA, 1 h at RT) and Alexa Fluor 588-phalloidin (Thermo Fisher/Invitrogen; 1:200 in PBS with 5 mg/ml BSA, 1 h at RT), as previously described [31, 36]. In the case of primary neurons, the Nrf2 antibody was co-incubated with mouse monoclonal anti-neurofilament 70 kDa antibody (clone DP5 2.7.3, Millipore-Sigma; 1:200) followed by immunoreaction with a combination of Alexa 488-labeled anti-rabbit and Alexa 588-conjugated anti-mouse antibodies (1:200, each; 1 h, RT). All images were acquired using a Zeiss AxioImager microscope and analyzed using ImageJ software.

#### Nuclear extract dot and Western blot analysis

The activation of Nrf2 following SH-SY5Y and primary neurons Aβ42 challenge—with or without MTZ or MEL co-treatment—was analyzed in nuclear cellular fractions by dot and Western blot assessing the nuclear translocation of the transcription factor. Preparation of subcellular nuclear fractions was performed as previously reported by our laboratory [31, 36], a methodology resulting in high-purity nuclear extracts as evidenced by the almost complete absence of Western blot signal for the cytoplasmic markers α-tubulin and GAPDH and the high positivity for nuclear histone 1. Briefly, after the different treatments, cells were collected in homogenization buffer [75 mM sucrose, 225 mM mannitol, 5 mM Tris-HCl pH 7.4, containing protease inhibitor cocktail (Roche Biochemical Reagents, Sigma)] and disrupted with the aid of a Dounce glass homogenizer. Cell homogenates were centrifuged (Eppendorf 5417R; 600×*g*, 5 min, 4 °C), nuclei recovered in the pellets, and lysed with RIPA buffer (Boston BioProducts, Ashland, MA); total protein content in the nuclear fractions was evaluated by BCA protein assay (Thermo Fisher Scientific/Pierce). For the dot blot assay, 20 μg of the respective nuclear extracts was loaded in the wells and blocked membranes probed with the rabbit polyclonal anti-Nrf2 antibody (1:500, overnight at 4 °C) followed by HRP-conjugated anti-rabbit IgG (GE Health Care Life Sciences; 1:5000, 1 h, RT) and ECL detection as above for Aβ oligomerization dot blot. For the Western blot evaluation of Nrf2 nuclear translocation, 20 μg each of the pertinent nuclear extracts was separated by 12% SDS-PAGE under reducing conditions and electrotransferred to 0.45 μm polyvinylidene difluoride membranes (PVDF, Thermo Fisher Scientific) for 1 h 45 min at 400 mA using 10 mM 3-cyclohexylamino-1-propanesulfonic acid (CAPS, Sigma) buffer, pH 11.0, containing 10% (v/v) methanol, as we previously described [31]. After blocking, the membranes were probed with anti-Nrf2 antibodies followed by HRP-conjugated anti-rabbit IgG, and finally developed by ECL, as for the dot blot. As loading controls for the dot and Western blot analyses, the membranes were stripped with Restore Plus Western blot stripping buffer (Thermo Fisher Scientific) and immunoreacted with rabbit polyclonal anti-GAPDH antibodies (Abcam, Cambridge, MA; 1:1000, overnight, 4 °C) followed by HRP-conjugated anti-rabbit IgG (GE Health Care Life Sciences; 1:5000, 1 h, RT).

#### Nrf2 activity ELISA

Active nuclear Nrf2 was evaluated using Nrf2 activity ELISA (Abcam) which quantitates Nrf2 able to bind to immobilized oligonucleotides containing the ARE consensus motif. Twenty micrograms of each of the nuclear protein extracts was loaded onto microtiter wells pre-coated with the specific double-stranded DNA sequence containing the Nrf2 consensus binding site (5′-GTCACAGTGACTCAGCAGAATCTG-3′). Active Nrf2 specifically captured by the oligonucleotide motif was detected by incubation with a primary antibody recognizing an Nrf2 epitope accessible only after protein activation and subsequent binding to its target DNA. This was followed by detection with HRP-conjugated secondary antibody and colorimetric readout at 450 nm in accordance with the manufacturer’s protocol.

### Evaluation of Nrf2-mediated antioxidant response

Changes in the activation of Nrf2 downstream antioxidant response were assessed through the immunocytochemical analysis of SOD1 and HO-1 in SH-SY5Y cells as well as in primary neurons grown on glass coverslips. After treatment with MTZ, MEL, and Trolox in the presence/absence of Aβ42, cells were fixed, and non-specific binding blocked as described above. This was followed by incubation with anti-SOD1 and anti-HO1 primary antibodies (Thermo Fischer/Invitrogen, 1:500 and 1:200, respectively, in PBS containing 5 mg/ml BSA, 2 h at RT) and subsequent reaction with the pertinent Alexa Fluor 488-conjugated anti-rabbit and anti-mouse IgG antibodies (Thermo Fisher/Invitrogen; 1:200 in PBS with 5 mg/ml BSA, 1 h at RT). The nuclei were counterstained with DAPI containing mounting medium (Verashield, Vector Laboratories, Burlingame, CA). Image acquisition and analysis were performed as above for the assessment of Nrf2.

### Assessment of methazolamide-, melatonin-, and Trolox-mediated Nrf2 activation pathway

Evaluation of the Nrf2 activation path elicited by MTZ, MEL, and Trolox was performed through assessment of the transcription factor nuclear translocation in the presence of PI3K and GSK-3 inhibitors, using as control cells incubated with SFN (5 μM), a compound capable of activating Nrf2 through disruption of its binding to Keap-1, a PI3K-independent pathway. SH-SY5Y cells plated on PDL-coated coverslips were challenged with the Nrf2 activators MTZ, MEL, Trolox, and SFN at the concentrations specified above in the presence of either the PI3K inhibitors LY294002 and Wortmannin (10 μM each) or the GSK-3 inhibitor SB216763 (10 μM) followed by evaluation of Nrf2 nuclear localization by immunocytochemistry as well as dot and Western blot analysis of nuclear extracts using the same procedures described above. Changes in the activation of Nrf2 downstream antioxidant response in the presence of PI3K and GSK-3 inhibitors was assessed through the immunocytochemical analysis of SOD1 and HO1, under identical conditions as above.

### Statistical analysis

Multiple comparison analyses were performed by ANOVA with Dunnett post hoc test using GraphPad (GraphPad, La Jolla, CA). Values of *p* ≤ 0.05 were considered significant.

## Results

### Aβ42 forms oligomeric assemblies in cell conditioned media

**Fig. 1:**
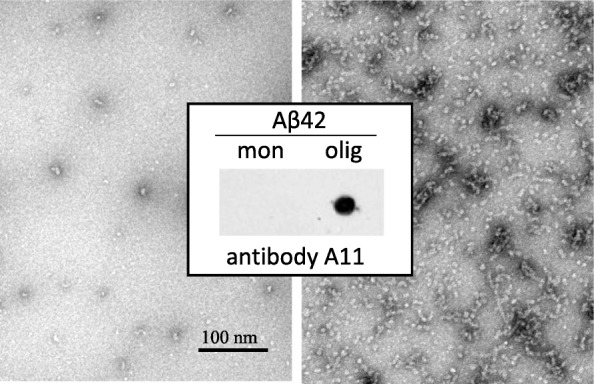
Aβ42 oligomeric structures visualized by dot blot and electron microscopy. The presence of oligomeric forms of Aβ42 in the culture supernatants was assessed by dot blot analysis probed with anti-oligomer antibody A11 as well as by electron microscopy after negative staining. Left panel: freshly solubilized HFIP-treated Aβ42; right panel: conditioned media of cells challenged with Aβ42 for 24 h. Bar, 100 nm

The formation of oligomeric assemblies during our experimental window was monitored by dot blot analysis probed with A11 anti-oligomer antibody whereas the final assessment of the conformational structures was evaluated by transmission electron microscopy. Figure 1 illustrates the characteristics of Aβ42 species in conditioned media of cells challenged with the peptide for 24 h, showing the presence of oligomeric structures of < 100 nm in length that were immunoreactive with the A11 antibody (right panel). As a control, freshly solubilized HFIP-treated Aβ42 lacked A11 immunoreactivity and failed to show similar structures under EM (left panel).

### Aβ42 induces mitochondria-mediated changes in neuronal metabolic profiles

**Fig. 2:**
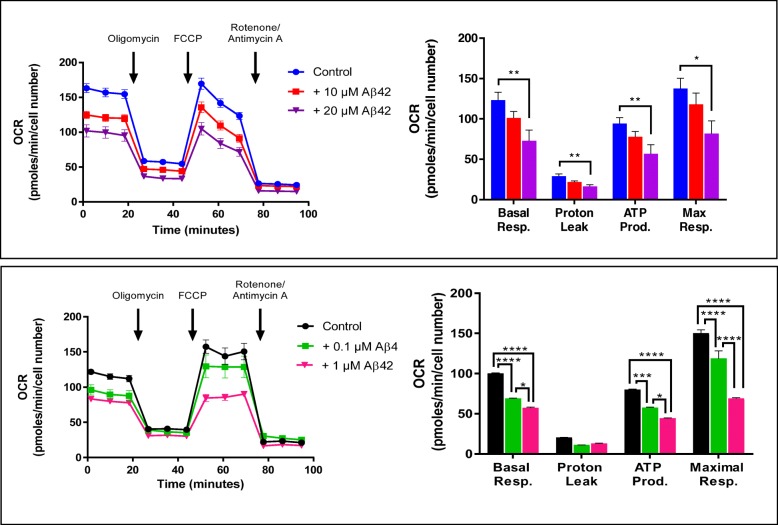
Aβ42-mediated metabolic/bioenergetic changes in SH-SY5Y cells and rat primary cortical neurons. Both cell types were incubated 24 h in the presence of increasing concentrations of Aβ42 and analyzed through the use of Cell Mito Stress Assay in a Seahorse platform as described in the “Material and methods” section. Panels on the left illustrate the real-time kinetic rate of oxygen consumption (OCR) versus time and indicate the injection time of the different modulators of the respiratory chain. Right panels depict the test parameters calculated with Seahorse XF Report Generator software. In all cases shown, OCR levels were normalized to DNA content assessed by Janus Green staining; bar graphs illustrate means ± SEM from at least three to six independent experiments performed in triplicate. *p < 0.05, **p < 0.01, ***p < 0.001, and ****p < 0.0001

Oxygen consumption by SH-SY5Y cells and fully differentiated DIV7 primary rat cortical neurons [39] was measured in the Seahorse XFe24 Extracellular Flux Analyzer which has become a reference method for analyzing mitochondrial function in intact cells. Basal respiration necessary to meet cellular energy demand is typically limited by the rate at which ATP is hydrolyzed, which in turn determines the rate of trans-mitochondrial membrane proton cycling into the matrix through ATP synthetase and out of the matrix through components of the electron transport chain. As shown in Fig. 2, baseline OCR values were stable in both cell types during the first 20 min. The addition of oligomycin decreased OCR in both cases to approximately the same extent − 25% and 20% of basal respiration levels for SHSY5Y and primary neurons, respectively—indicating low levels of oligomycin-resistant OCR attributable to proton leakage. Subsequent addition of the proton ionophore FCCP elicits the maximal rate at which O_2_ is consumed. The compound collapses proton electrochemical gradients permitting the maximal functioning of the respiratory chain without limitation by a positive outside membrane potential. As a result, FCCP uncouples respiration from ATP synthesis allowing determination of the maximal respiratory capacity of the electron transport chain only limited by substrate supply. In our system, FCCP completely restored basal respiration levels in SH-SY5Y cells and increased OCR values in ~ 60% in DIV7 neurons, consistent with previous findings [39, 40]. The difference in the FCCP response between SH-SY5Y and primary neurons likely reflects neuronal differentiation, as it has been reported that while maximal respiration did not increase above basal levels in non-differentiated cells, FCCP-induced OCR values were significantly higher in differentiated neurons [39]. At the final steps of the Seahorse run, the addition of the respiratory complex I inhibitor rotenone in combination with the complex III inhibitor antimycin allowed evaluation of non-mitochondrial OCR, an index of oxygen-consuming processes unrelated to the mitochondrial activity. In our system, the addition of these inhibitors resulted in > 90% inhibition of OCR, confirming that for both SH-SY5Y and primary neurons, the levels of O_2_ consumption are almost exclusively related to mitochondrial respiration, following the trend observed in most cell types [41].

Incubation with Aβ42 at concentrations and in a time frame in which the peptide does not induce cell toxicity [7, 35, 42] changed the bioenergetic profiles of both SH-SY5Y and primary neuron cells in a dose-dependent manner, albeit it should be noted that primary neurons were more susceptible to the effect of the peptide with comparable responses elicited at much lower doses. Figure 2 illustrates the Aβ-induced changes in the real-time metabolic parameters. Aβ42 caused a significant reduction in basal respiration calculated by subtracting non-mitochondrial respiration—OCR values after antimycin A/rotenone injection—from baseline respiration levels (Additional file 1: Figure S1). In SH-SY5Y cells (Fig. 2, panel A) basal OCR levels reached ~ 54% of OCR control values after incubation with 20 μM Aβ42, the highest concentration tested in our system since higher Aβ doses caused significant cell death [31, 35]. Notably, a comparable 55% OCR reduction was elicited in primary neurons (panel B) by 1 μM Aβ42, a 20-fold lower concentration. In addition, Aβ42 significantly decreased maximal respiration calculated by subtraction of non-mitochondrial respiration values from the maximum respiration levels induced by injection of FCCP (Additional file 1: Figure S1). As illustrated in Fig. 2, SH-SY5Y maximal respiration was reduced by incubation with 20 μM Aβ42 to levels ~ 55% of those in untreated control cells. In the case of primary neurons, a much lower 1 μM Aβ42 concentration decreased OCR to ~ 39% of untreated control levels. Another metabolic parameter affected by Aβ is the proton leak, calculated by subtracting non-mitochondrial OCR values from respiration levels in the presence of the ATP synthase inhibitor oligomycin (Additional file 1: Figure S1). In both neuronal cells, the low proton leak levels observed (~ 10% of basal respiration) indicate that O_2_ consumption is primarily coupled to ATP production. The addition of Aβ42 appears to reduce these values even more likely indicating that the damage to oxidative phosphorylation induced by the peptide impedes the flow of electrons thereby resulting in even lower OCR values. Aβ42 also caused a significant decrease in O_2_ consumption linked to ATP production which is evaluated in the metabolic analyzer as the difference between basal and oligomycin-insensitive OCR measurements (Additional file 1: Figure S1). As with the other metabolic parameters, incubation with Aβ42 induced a similar reduction in ATP production in primary neurons albeit at 20-fold lower doses than in SH-SY5Y (~ 60% reduced values in 1 μM-treated primary neurons versus ~ 56% of control values in SH-SY5Y challenged with 20 μM Aβ42).

Overall, Aβ42 caused a significant decrease in all Seahorse-assessed metabolic parameters in SH-SY5Y and primary neurons although the latter were more susceptible to the effect of the peptide and comparable changes were notable at much lower doses. Whether these differences relate to the differentiation state of the cells remains to be elucidated, albeit it has been previously demonstrated during ex vivo differentiation of primary cortical neurons that neuronal development significantly modifies mitochondrial activity and global metabolic parameters [39].

### Methazolamide, melatonin, and Trolox rescue Aβ42-induced changes in neuronal metabolic/bioenergetic profiles

**Fig. 3:**
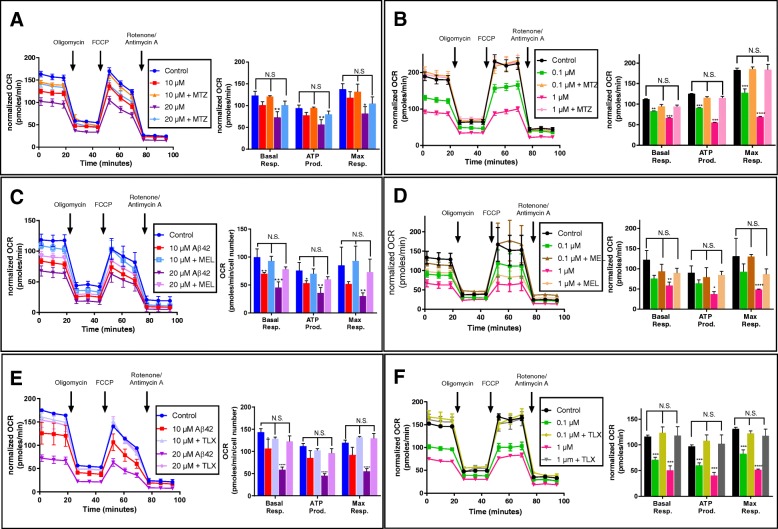
Methazolamide, melatonin, and Trolox prevent Aβ42-induced mitochondrial dysfunction in human SH-SY5Y cells and rat primary cortical neurons. Both cells were grown in Seahorse XFe24 culture plates, incubated 24 h with increasing concentrations of Aβ42 in the presence or absence of MTZ (for SH-SY5Y (**a**) and primary neurons (**b**)), MEL (for SH-SY5Y (**c**) and primary neurons (**d**)), and Trolox (for SH-SY5Y (**e**) and primary neurons (**f**)). Cell bioenergetic profiles were analyzed with Cell Mito Stress Assay in a Seahorse XFe24 metabolic analyzer. Panels on the left illustrate the real-time kinetic rate of oxygen consumption (OCR) and indicate the injection time of the different respiratory chain modulators; right panels depict the test parameters calculated with Report Generator software. In all cases shown, OCR levels were normalized to DNA content assessed by Janus Green staining; bars illustrate means ± SEM from at least three to six independent experiments performed in triplicate. *p < 0.05, **p < 0.01, ***p < 0.001, and ****p < 0.0001

Previous work from our laboratory as well as from other groups has demonstrated a protective effect of a number of compounds on Aβ-induced cellular toxicity and mitochondrial activity, among them MTZ, MEL, and Trolox [8, 24, 26, 35, 43]. These three compounds were tested for their ability to circumvent the effect of Aβ42 in the metabolic parameters evaluated in the Seahorse platform at a concentration previously shown as protective from Aβ-mediated mitochondrial cytochrome c release and induction of apoptosis, a dosage at which the compounds did not exhibit toxicity [8, 23, 24, 26, 35, 43]. Figure 3 illustrates the metabolic profile of Aβ42-challenged SH-SY5Y and primary cortical neurons (panels a and b, respectively) in the presence of a 300-μM dose of MTZ, a concentration that in our previous work protected SH-SY5Y cells from Aβ-mediated mitochondrial cytochrome c release and prevented downstream activation of caspase-3 and subsequent apoptosis [24, 31]. As depicted in the figure, MTZ rescues the metabolic/bioenergetic deficits induced by the peptide, restoring basal and maximal respiration as well as ATP production to the levels of untreated SH-SY5Y and cortical neurons controls.

Figure 3 also illustrates the protective effect of MEL in circumventing the detrimental effect of Aβ42 in neuronal cells. The decrease in all mitochondria-mediated metabolic parameters observed in Aβ-challenged SH-SY5Y and cortical neurons (panels c and d, respectively) was restored to control levels by treatment with 100 μM MEL, a non-toxic concentration in the range of previously reported antioxidant activity of the compound [22, 23, 26, 44]. Basal and maximal respiration, as well as O_2_ consumption linked to ATP production, were all restored to levels non-statistically different from those of non-Aβ-treated neurons. The protective effect of MTZ and MEL in neuronal metabolic profiles was also recapitulated by treatment with Trolox, a vitamin E analog with high ROS-scavenging capacity that is used as a standard for the evaluation of the antioxidant capability of other compounds [45, 46]. As illustrated in Fig. 3e, f, co-incubation of Aβ42-challenged SH-SY5Y cells and cortical neurons with 300 μM Trolox, a dose capable of protecting cells from amyloid-mediated ROS generation [35, 36], rescued the decrease induced by the Aβ peptide in basal and maximal respiration, as well as ATP production levels. The protective effect of MTZ, MEL, and Trolox likely results from the capability of the compounds to increase mitochondrial function in the Seahorse platform as indicated by their ability to increase basal and maximal respiration when incubated 24 h with SH-SY5Y cells in the absence of Aβ (not shown).

### Methazolamide, melatonin, and Trolox protect neuronal cells from the Aβ-mediated generation of reactive oxygen species

**Fig. 4:**
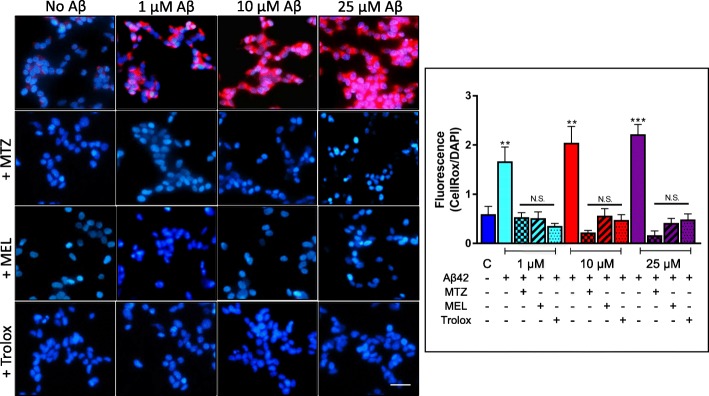
Methazolamide, melatonin, and Trolox protect from Aβ-mediated ROS generation in SH-SY5Y. Following 24 h incubation with Aβ42 (0–25 μM) in the presence or absence of MTZ (300 μM), MEL (100 μM), and Trolox (300 μM), ROS-generated species were detected with CellROX (5 μM), and nuclei counterstained with Hoechst (1 μg/ml). Images depict CellRox fluorescence (red signal) and DAPI DNA counterstaining; bar, 25 μm. The graph on the right illustrates the quantitation of CellROX fluorescence values normalized to DAPI signal using ImageJ analysis software; data is represented as mean ± SEM. **p < 0.01 and ***p < 0.001

**Fig. 5:**
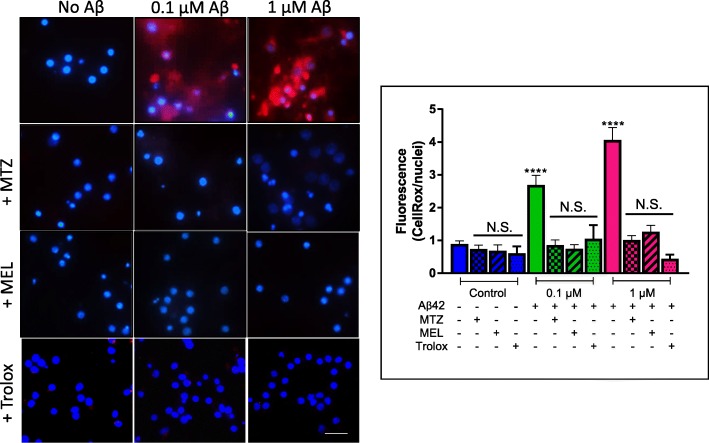
Methazolamide, melatonin, and Trolox prevent Aβ-mediated ROS generation in primary cortical neurons. Primary neurons were treated with various concentrations of Aβ42 (0–1 μM) in the presence or absence of MTZ (300 μM), MEL (100 μM), and Trolox (300 μM), subsequently stained with CellROX, and nuclei counterstained with Hoechst, as in Fig. 4. Immunofluorescence images illustrate ROS formation highlighted by the free radical sensing probe CellROX (red signal) and nuclei staining with DAPI (blue fluorescence); bar, 25 μm. The graph depicts the quantitation of CellROX fluorescence normalized to DAPI signal with ImageJ software; data is represented as mean ± SEM. ****p < 0.0001

Oxidative stress, often linked to mitochondrial dysfunction, is integral to many cell death programs and has been associated with the progression of a number of neurodegenerative disorders [47, 48]. SH-SY5Y cells and primary cortical neurons challenged 24 h with increasing concentrations of Aβ42 (0–25 μM and 0–1 μM, respectively) displayed a progressive generation of reactive oxygen species (ROS), as highlighted by the free radical sensing fluorogenic probe CellROX (Figs. 4 and 5, respectively). This ROS generation was completely blocked by treatment with the protective agents MTZ, MEL, and Trolox at a concentration at which the compounds do not exhibit cell toxicity. The bar graphs in each figure illustrating the quantitation of the red fluorescent signal, normalized to DAPI staining intensity, clearly indicate the dose response of ROS buildup with increasing concentrations of the peptide, as well as the complete inhibition of ROS generation in the presence of MTZ, MEL, and Trolox all of which bring back levels to values that do not statistically differ from those of non-Aβ-treated control cells. Whether the compounds are capable of reversing Aβ-induced changes, once the detrimental effect of the peptide has occurred, remains to be elucidated.

### Effect of methazolamide, melatonin, and Trolox on Aβ oligomerization

**Fig. 6:**
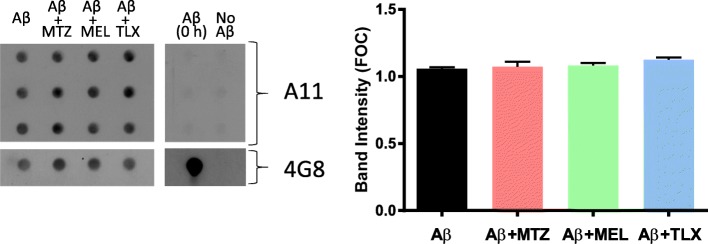
Lack of effect of methazolamide, melatonin, and Trolox on Aβ oligomerization. Oligomer Aβ immunoreactivity in cell culture supernatants from SH-SY5Y cells treated 24 h with Aβ42 (10 μM) in the presence/absence of MTZ (300 μM), MEL (100 μM), or Trolox (300 μM) was assessed by dot blot analysis. The respective supernatants of triplicate experiments were loaded onto the nitrocellulose membrane and probed with A11 anti-oligomer antibody, as described in the “Material and methods” section. Reactivity with 4G8 anti-Aβ antibody was used as a loading control. Right panel depicts the immunoreactivity of membranes loaded with freshly prepared synthetic Aβ42 (800 ng) as a negative control for oligomer formation. Histograms on the right panel illustrate the densitometric quantification of the dot blot A11 signal intensities normalized to the respective 4G8 controls; data are represented as mean ± SEM

Our previous reports as well as work from other laboratories have demonstrated the key role exerted by Aβ oligomerization in the induction of mitochondria-mediated cell death pathways [7, 8, 24, 31, 35]. To assess whether the protective effect of MTZ, MEL, and Trolox on the Aβ-mediated metabolic, bioenergetic, and oxidative changes illustrated above resulted from changes in Aβ oligomerization induced by the compounds, we performed dot blot analysis using the anti-Aβ oligomer A11 antibody. In the assay, the conditioned media of triplicate experiments in which SH-SY5Y cells challenged 24 h with Aβ42 in the presence and absence of MTZ, MEL, and Trolox were probed with the anti-oligomer antibody. As illustrated in Fig. 6, no changes in the oligomer signal intensity were observed upon treatment with the protective compounds under the current experimental conditions. As a control, and consistent with previous reports [8], the figure highlights the lack of A11 reactivity of freshly solubilized HFIP-treated Aβ42 while reactivity with monoclonal 4G8 anti-Aβ antibody was used as a loading control.

### Methazolamide, melatonin, and Trolox activate Nrf2 transcription factor and the downstream antioxidant response

**Fig. 7:**
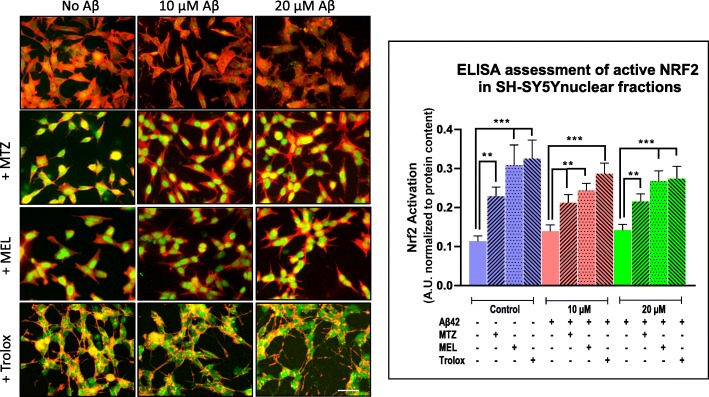
Methazolamide, melatonin, and Trolox activate Nrf2 nuclear translocation in SH-SY5Y cells. SH-SY5Y cells were treated with various concentrations of Aβ42 (0–20 μM) in the presence/absence of MTZ, MEL, or Trolox. Nrf2 nuclear translocation was assessed by incubation with rabbit polyclonal anti-Nrf2 antibody followed by Alexa-488 conjugated anti-rabbit IgG, as detailed in the “Materials and methods” section. Actin was highlighted by immunoreaction with Alexa-588 conjugated phalloidin. Immunofluorescence images depict Nrf2 nuclear signal (green fluorescence) and actin staining (red signal); bar, 25 μm. The graph illustrates the Nrf2 activity in nuclear extracts evaluated by ELISA through the quantitation of Nrf2 binding to immobilized oligonucleotides containing the ARE consensus motif. Bars represent mean ± SEM of triplicate experiments. **p < 0.01 and ***p < 0.001

**Fig. 8:**
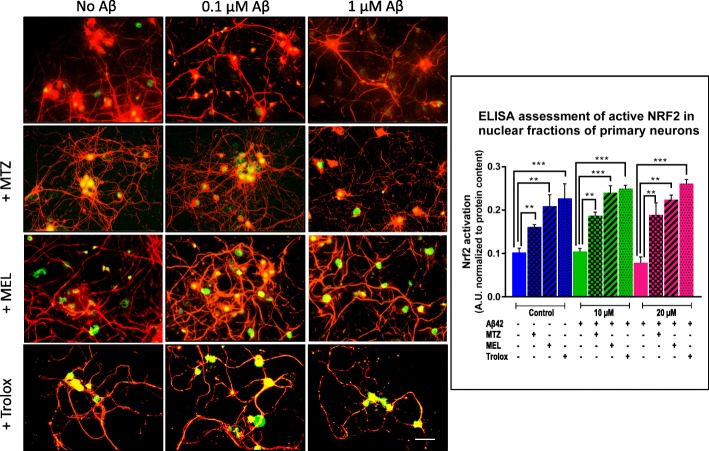
Methazolamide, melatonin, and Trolox induce Nrf2 activation in primary neurons. Cortical neuron cultures were challenged with various concentrations of Aβ42 (0–1 μM) in the presence or absence of either MTZ (300 μM), MEL (100 μM), or Trolox (300 μM) and Nrf2 assessed by immunofluorescence microscopy as in Fig. 7. Immunofluorescence images depict Nrf2 nuclear translocation (green signal) and neurofilament protein staining (red fluorescence); bar, 25 μm in all images. The graph illustrates the Nrf2 activity in nuclear extracts evaluated by ELISA. Bars represent mean ± SEM of triplicate experiments. **p < 0.01 and ***p < 0.001

Based on the lack of effect of MTZ, MEL, and Trolox on Aβ oligomerization, we looked into whether the protective effects of the compounds resulted from activation of Nrf2, a central regulator of the antioxidant response [49, 50]. As the first step, we evaluated the nuclear translocation of the transcription factor induced by the compounds in SH-SY5Y and primary neurons by immunocytochemistry. Figures 7 and 8 illustrate the increase in the nuclear Nrf2 green fluorescent signal induced by MTZ, MEL, and Trolox in both cell types in comparison with the low endogenous levels of expression present in untreated control cells. Notably, the addition of Aβ to the cells did not result in Nrf2 activation at any of the Aβ concentrations tested in spite of the ROS-inducing activity of the peptide illustrated in Figs. 4 and 5. The Nrf2 nuclear translocation observed following MTZ, MEL, and Trolox treatment of Aβ-challenged cells suggests that the protective action of the compounds from the detrimental effect of the peptide is likely driven by the induction of Nrf2-mediated antioxidant response defense mechanisms.

**Fig. 9:**
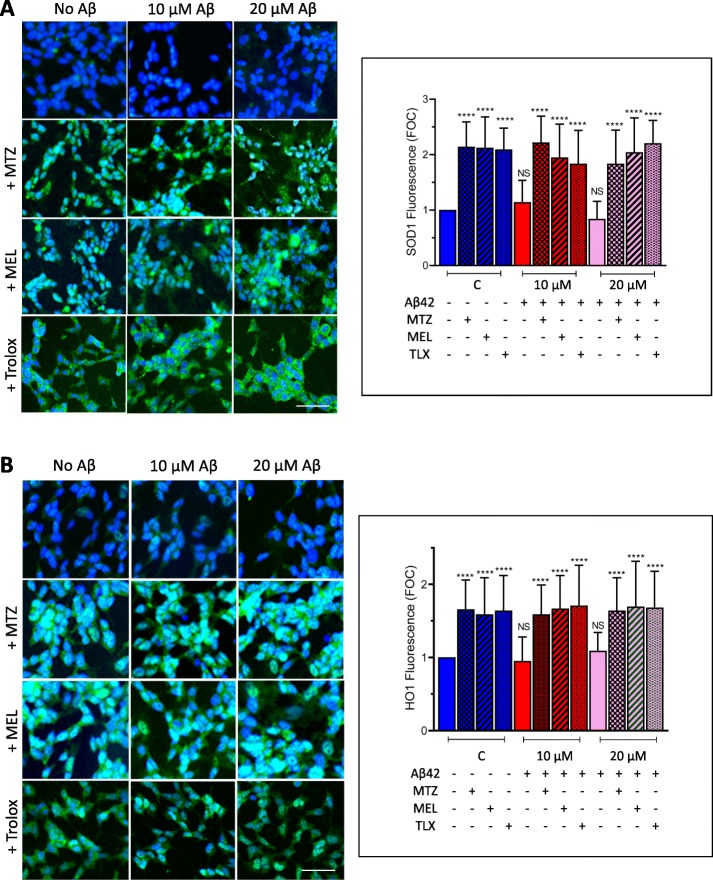
Translational activation of the downstream Nrf2 targets SOD-1 and HO-1 by methazolamide, melatonin, and Trolox in SH-SY5Y cultures. Cells were treated with the Nrf2 activators MTZ, MEL, and Trolox in the presence/absence of Aβ42 (0–20 μM) followed by immunodetection of SOD1 and HO-1 expression by incubation with the pertinent primary antibodies and subsequent immunoreaction with Alexa-488 conjugated secondary antibodies. Green fluorescence highlights SOD-1 (**a**) and HO-1 (**b**) immunoreactivity; blue fluorescence depicts DAPI nuclear DNA counterstaining. Bar, 25 μm in all cases. The graphs in **a** and **b** depict respectively the quantitation of SOD-1 and HO-1 fluorescence signal in at least 400 cells utilizing ImageJ software and expressed in fold of control. Data is represented as mean ± SD; ****p < 0.0001

**Fig. 10:**
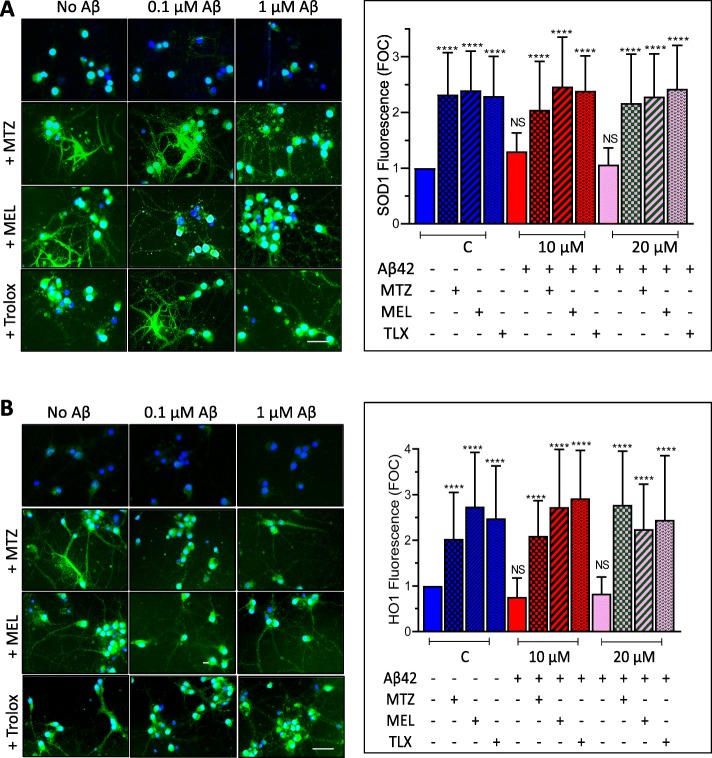
Translational activation of the downstream Nrf2 targets SOD-1 and HO-1 by methazolamide, melatonin, and Trolox in primary cortical neurons. Cells were treated with the Nrf2 activators MTZ, MEL, and Trolox in the presence/absence of Aβ42 (0–1 μM) as in Fig. 9. Expression of SOD-1 (**a**) and HO-1 (**b**) was detected by immunocytochemical analysis using rabbit polyclonal anti-SOD-1 and mouse monoclonal anti-HO-1 primary antibodies followed by the pertinent Alexa-488 conjugated secondary antibodies. Green fluorescence highlights SOD-1 (**a**) and HO-1 (**b**) immunoreactivity; blue fluorescence depicts DAPI nuclear DNA counterstaining. Bar, 25 μm in all images. The graphs in **a** and **b** depict respectively the quantitation of SOD-1 and HO-1 fluorescence signal in 50–100 cells utilizing ImageJ software and expressed in fold of control. Data is represented as mean ± SD; ****p < 0.0001

Confirmation of Nrf2 activation by MTZ, MEL, and Trolox was assessed by Nrf2 activity ELISA, a test with a lower detection limit of 0.6 μg nuclear extract/well. After a separate treatment of Aβ-challenged SH-SY5Y and primary neurons with the different compounds and preparation of nuclear extracts, the capability of the transcription factor in these fractions to bind the ARE motif was evaluated through Nrf2 ELISA activity assay following the protocols described in the “Material and methods” section. As illustrated in Fig. 7, right panel, nuclear active Nrf2 more than doubled with the addition of MTZ, MEL, and Trolox to control non-Aβ-treated SH-SY5Y cells. The enhanced nuclear expression of Nrf2 caused by the compounds was also observed in the presence of Aβ42, likely accounting for their protective effect from Aβ42 described above. MTZ, MEL, and Trolox exhibited a comparable effect in enhancing the levels of nuclear active Nrf2 in primary cortical neurons (Fig. 8, right panel). The activation of Nrf2 elicited by the three compounds in both Aβ- and non-Aβ-challenged SH-SY5Y cells and primary neurons translated, in all cases, in downstream increased expression of SOD-1 and HO-1, as illustrated by the immunocytochemical evaluation of both enzymes. Figure 9 illustrates the cytoplasmic increment of SOD-1 (Fig. 9a) and HO-1 expression (Fig. 9b) following 24 h incubation with MTZ, MEL, and Trolox in SH-SY5Y. All compounds produced statistically significant differences of about 2-fold increase in the levels of cytoplasmic SOD-1 signal and ~ 1.5-fold increase in HO-1 fluorescence intensity compared to untreated control cells as evaluated using ImageJ software. Similarly, Fig. 10a, b depicts a comparable effect of MTZ, MEL, and Trolox in primary cortical neurons. All compounds produced a statistically significant difference of ~ 2 to 2.5-fold increase in the levels of cytoplasmic SOD-1 and HO-1 green fluorescent signal compared to untreated control primary neurons.

### Methazolamide, melatonin, and Trolox activate Nrf2 and its downstream antioxidant response proteins through the PI3K/GSK-3 axis

**Fig. 11:**
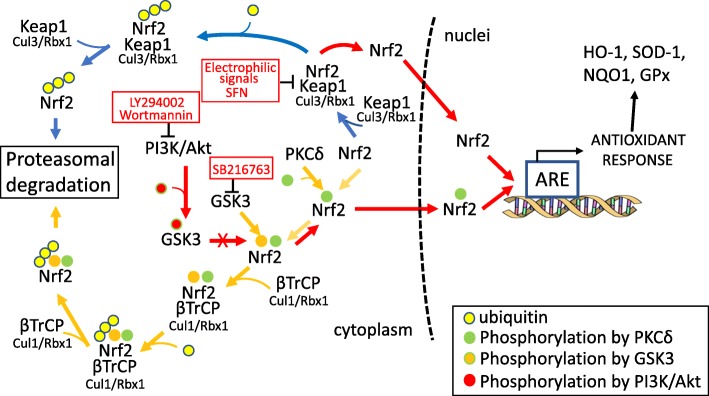
The main activation pathways of Nrf2. The diagram illustrates the main homeostatic pathways regulating the transcription factor Nrf2 proteosomal degradation and activation through its nuclear translocation. The figure indicates the sites of action of PI3K inhibitors LY294002 and Wortmannin, the GSK-3 inhibitor SB216763, as well as SFN, an electrophilic activator that disrupts the Nrf2-Keap1complex formation

**Fig. 12:**
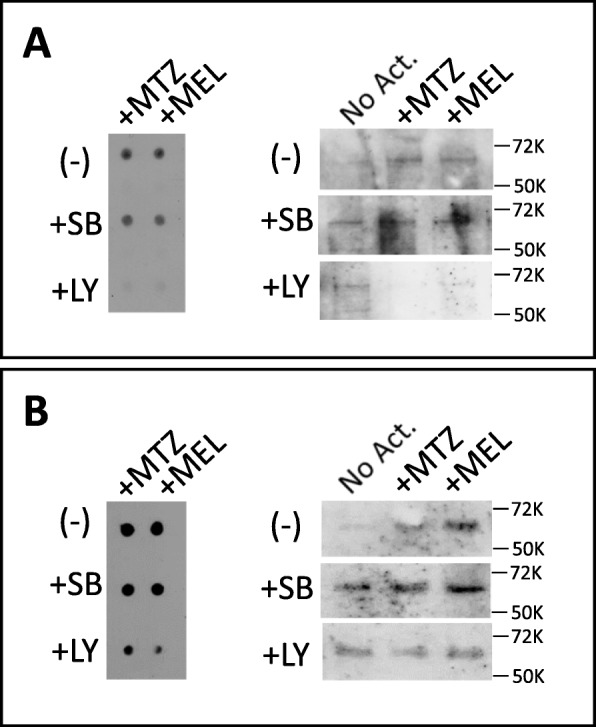
Assessment of PI3K-mediated Nrf2 activation in SH-SY5Y and primary neurons by dot and Western blot analyses. SH-SY5Y cells and primary cortical neurons were treated with MTZ (300 μM) and MEL (100 μM) in the presence/absence of the PI3K inhibitor LY294002 and the GSK-3 inhibitor SB216763 (both at 10 μM concentration). This was followed by an evaluation of Nrf2 reactivity in nuclear extracts by dot and Western blot analysis following the protocols described in the “Material and methods” section. **a** SH-SY5Y cells. **b** Primary neurons. In both cases, left panels illustrate the dot blot images and right panels the Western blot detection

Figure 11 illustrates the two major pathways of Nrf2 degradation and activation, one mediated by its binding to Keap1 (Kelch-like ECH-associated protein 1) and the other regulated by glycogen synthase kinase 3 (GSK-3). Nrf2 activation was evaluated assessing its nuclear translocation and subsequent triggering of the antioxidant proteins SOD1 and HO-1 in SH-SY5Y cells and primary neurons incubated with MTZ, MEL, and Trolox in the presence/absence of PI3K and GSK-3 inhibitors. As illustrated in Fig. 12, MTZ and MEL challenge of SH-SY5Y cells and primary neurons in the presence of the PI3K inhibitor LY294002, almost completely abolished the dot and WB Nrf2 signal in the respective nuclear fractions suggesting that the activation of Nrf2 by the two compounds takes place through the PI3K/Akt axis. Additional corroboration was obtained with the use of the GSK-3 inhibitor SB216763. Under normal conditions, GSK-3 targets Nrf2 for proteosomal degradation through its phosphorylation and subsequent binding to the βTrCP E3 ubiquitin ligase complex (Fig. 11). GSK-3 inhibition precluded Nrf2 degradation resulting in its enhanced expression and nuclear translocation evidenced by the dot and WB signal intensities (Fig. 12), further implicating the PI3k/Akt-GSK-3 axis in the activation pathway elicited by MTZ and MEL.

**Fig. 13:**
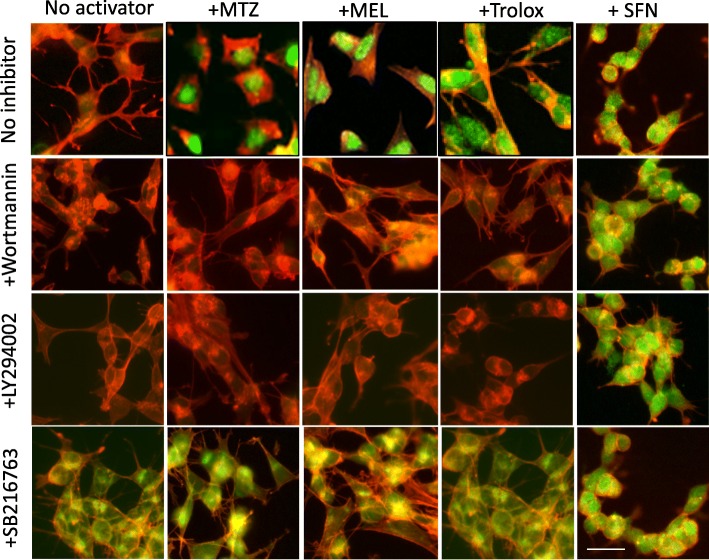
Methazolamide, melatonin, and Trolox activate Nrf2 through a PI3K-mediated pathway. SH-SY5Y cells were treated with MTZ (300 μM), MEL (100 μM), or Trolox (300 μM) in the presence of the PI3K inhibitors LY294002 and Wortmannin (10 μM each) or the GSK-3 inhibitor SB216763 (10 μM). As a control, cells were incubated with SFN (5 μM), a compound capable of activating Nrf2 through disruption of its binding to Keap-1, a PI3K-independent pathway. In all cases, Nrf2 expression was evaluated by immunocytochemistry as in Figs. 7 and 8. Green fluorescence highlights Nrf2 nuclear translocation, and red fluorescence depicts actin staining with Alexa 588-conjugated phalloidin. Bar represents 20 μm in all images. Quantitation of the nuclear fluorescence signal is shown in Additional file 2: Figure S2

Validation of Nrf2 nuclear translocation and concomitant increase in the expression of the antioxidant proteins SOD1, and HO-1 was performed by immunocytochemistry in SH-SY5Y cells incubated with MTZ, MEL, and Trolox as well as with the electrophilic compound SFN, in the presence/absence of PI3K and GSK-3 inhibitors. As illustrated by the images in Fig. 13 and their quantitative assessment in Additional file 2: Figure S2, the addition of the PI3K inhibitors Wortmannin and LY294002 resulted in complete inhibition of Nrf2 activation as evidenced by the lack of nuclear fluorescence signal strongly suggesting that the activation of Nrf2 by MTZ, MEL, and Trolox takes place through the PI3K/Akt-GSK-3 axis. Additional corroboration was obtained with the use of the GSK-3 inhibitor SB216763. GSK-3 inhibition precluded Nrf2 degradation through the β-TrCP path resulting in its enhanced nuclear expression, further implicating the PI3k/Akt-GSK-3 axis in the activation mechanism elicited by MTZ, MEL, and Trolox (Fig. 13). In contrast, cells challenged with SFN—an electrophilic compound known to activate Nrf2 through disruption of the binding to its transport protein Keap1, a PI3K-independent path—were not affected by the addition of the PI3K inhibitors and retained the intense nuclear Nrf2 immunostaining.

**Fig. 14:**
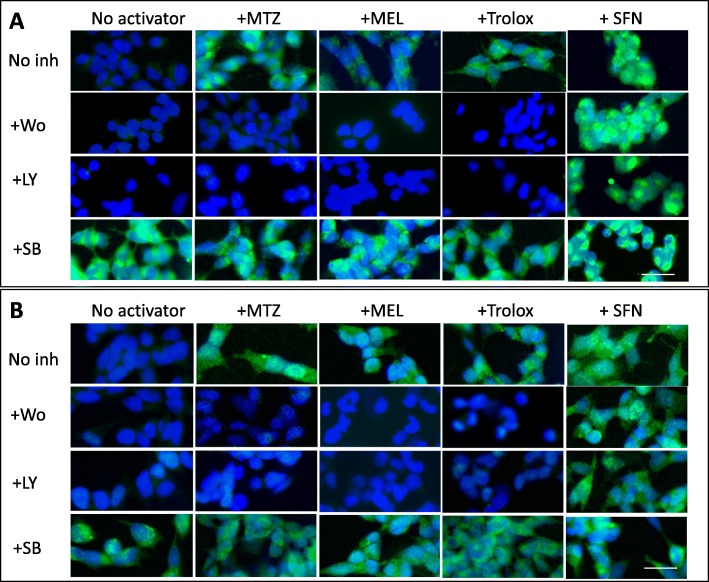
Methazolamide, melatonin, and Trolox activate Nrf2 downstream antioxidative response proteins SOD-1 and HO-1 through the PI3K axis. SH-SY5Y cells were treated with MTZ, MEL, Trolox, or SFN in the presence of the PI3K inhibitors LY294002 and Wortmannin or the GSK-3 inhibitor SB216763 as in Fig. 13. In all cases, SOD-1 and HO-1 expression was evaluated by immunocytochemistry using the respective primary antibodies followed by Alexa 488-conjugated secondary antibodies. Green fluorescence highlights SOD-1 (**a**) and HO-1 (**b**) reactivity; blue fluorescence depicts DAPI nuclear DNA counterstaining. Bar, 25 μm in all images. Quantitation of the cytoplasmic SOD-1 and HO-1 fluorescent signals is shown in Additional file 3: Figure S3

The MTZ-, MEL-, and Trolox-mediated activation of the antioxidant response proteins SOD1 and HO-1 was studied in the presence of Wortmannin, LY294002, and SB216763. Consistent with the effect of the different inhibitors on the nuclear Nrf2 translocation, Fig. 14 depicts representative images of the PI3K-dependence of SOD1 and HO-1 activation and the lack of effect of the GSK-3 inhibitor, whereas Additional file 3: Figure S3 shows the corresponding quantitative evaluation of the images. The four- to six-fold increments in fluorescence values induced by the presence of MTZ, MEL, and Trolox were brought down to levels not significantly different than untreated control cells in the presence of PI3K inhibitors whereas the inclusion of the GSK-3 inhibitor SB216763 had no effect. As anticipated, the PI3K-independent SFN-mediated activation of Nrf2 downstream proteins was not affected by the Wortmannin and LY294002 inhibitors. Overall, the data indicates that MTZ, MEL, and Trolox activate the cellular antioxidant response via the PI3K/Akt-GSK-3 axis.

## Discussion

The brain accounts for only ~ 2% of the total body mass but receives up to 20% of cardiac output and is responsible for 20–25% of the body’s O_2_ and glucose consumption, highlighting the high energy requirement essential for the maintenance of its physiological functions [51]. As the brain has limited energy storage capacity, mitochondria are crucial organelles for generating through OXPHOS the levels required to avoid even brief periods of energy deprivation that would result in cell dysfunction and death. These essential organelles—primarily abundant in cells with high energy demands, as neurons—control cell bioenergetics and ROS homeostasis with recent data stressing their critical role in the regulation of the blood-brain barrier permeability and synaptic integrity, actively participating in the underlying mechanisms of learning and memory [52–54].

Dysregulation of mitochondrial downstream pathways in AD brains has been demonstrated in multiple studies [55–58]. Numerous investigations have illustrated increased generation of free radicals, lipid peroxidation, oxidative DNA, and protein damage in conjunction with decreased ATP production [59–63]. Altered cerebral glucose utilization, a metabolic feature tightly related to mitochondrial function [59] and an invariant AD pathophysiological feature, is in fact increasingly recognized as a critical contributor to disease pathogenesis [64, 65]. Impairment of glucose metabolism, particularly in areas with dense synaptic content, was demonstrated by [^18^F]-fluorodeoxyglucose positron emission tomography ([^18^F] FDG-PET) [66] and magnetic resonance spectroscopy (MRS) [64] approaches. Biochemical analyses have shown decreased activity of key enzymes controlling metabolic flux to the tricarboxylic acid (TCA) cycle—pyruvate dehydrogenase, α-ketoglutarate dehydrogenase, and isocitrate dehydrogenase—as well as changes in malic enzyme, a major anaplerotic component in neurons, parameters correlating with cognitive impairment [67–72]. Most of these findings were also recapitulated in different rodent transgenic lines including the widely studied Tg2576 [73–80]. In this model, caspase-3 activity was found elevated in hippocampal synapsis before amyloid deposition was detectable, causing reduction of spine density in CA1 pyramidal neurons, alterations in glutamatergic synaptic transmission, and deficits in hippocampal-dependent contextual fear conditioning [52], all features rescued by pharmacologic inhibition of caspase-3 and suggestive of the central role of mitochondria in early synaptic dysfunction. Further highlighting mitochondrial dysfunction in this model, proteomic analysis has shown early dysregulation in mitochondrial proteins [81] while metabolomic profiling indicated alterations in energy metabolism [82]. Thus, targeting early synaptic deficits by halting the detrimental effects of oligAβ on mitochondrial dysfunction and synaptic integrity has emerged as potentially effective—although still elusive—strategies to preserve cognitive function [83].

A common feature in the progression toward cell death is the release of cytochrome c from the mitochondria into the cytoplasm leading to the formation of the apoptosome and downstream activation of terminal caspase-3 [84, 85]. Almost a decade ago, MTZ and MEL were identified as two drugs capable of preventing cytochrome c release in isolated mitochondria and exhibiting neuroprotective properties in models of ischemic injury and Huntington disease [22, 23]. More recent work from our lab has studied the effect of MTZ on Aβ-mediated mitochondrial dysfunction in different cell culture models demonstrating the ability of the compound in protecting cells from the detrimental effect of Aβ, preventing the Aβ-elicited release of cytochrome c and changes in the organelle membrane potential, and ultimately inhibiting the induction of cell death mechanisms [7, 8, 24]. The work presented herein demonstrate that both MTZ and MEL, as well as the potent ROS scavenger and vitamin E analog Trolox, not only prevent the formation of oxidative radicals in SH-SY5Y and primary cortical neurons but also protect from the metabolic/bioenergetic deficits induced by Aβ, restoring basal and maximal respiration as well as ATP production to the levels of untreated cells. Whether the protective effect of MTZ, MEL, and Trolox on the Aβ-mediated metabolic/bioenergetic alterations results from the ROS-scavenging activity of the compounds and reflects the intricate relationship between ROS homeostasis and respiration [86] remains to be determined.

In spite of the comparable protective effect exerted by MTZ, MEL, and Trolox in the neuronal Aβ-mediated changes observed in our in vitro paradigm, all three compounds have a strikingly different described primary activity. MTZ, FDA-approved for the local treatment of glaucoma, has been reported to act as a carbonic anhydrase inhibitor and as such modulates the reversible conversion of CO_2_ to HCO3^−^ essential for the anaplerotic replenishing of TCA intermediates and for the regulation of carboxylating enzymes using CO_2_ as substrate, being among them malic enzyme, propionyl-, methylcrotonyl-, and acetyl-CoA carboxylases the most relevant in neurons [87]. MEL is a pineal gland hormone that regulates circadian rhythms of physiologic activities as sleep [22]. It is a potent antioxidant active in different in vivo systems, including the CNS and at the level of the synapses [29, 88] but does not exhibit recognizable carbonic anhydrase inhibitory activity. More recent work has demonstrated a more wide-ranging effect of MEL on mitochondrial activity precluding astrocytic Aβ-mediated mitochondrial depolarization [88] as well as preventing caspase-3-mediated apoptosis and enhancing ATP synthesis under conditions of metabolic- and radiation-mediated stress [28, 89]. Trolox is a water-soluble vitamin E analog which due to its high capacity to capture ROS is used as a standard for the evaluation of the antioxidant capacity of other molecules [45]. Through its capacity to quench and react with single oxygen and neutralize free radical species, which indeed surpasses that of vitamin E [90, 91], Trolox has been reported to prevent neurotoxicity induced by Aβ and hydrogen peroxide [92, 93].

The dissimilar primary biological activities of MTZ, MEL, and Trolox provide an interesting paradigm to better dissect how potentially converging pathways are involved in the Aβ-induced mitochondrial dysfunction described in the current work. Based on the reports of its downregulation in Alzheimer’s patients and transgenic models described below, as well as in its wide role in regulating metabolic pathways compromised in the disease, we focused our attention in the central regulator of the antioxidant response, the nuclear factor erythroid 2-related factor 2 (Nrf2) [49, 50], a redox state-dependent transcription factor and key regulator of inducible defense systems [94]. Located in the cytosol with constitutive low levels strictly controlled by the proteasome [95], its degradation is drastically reduced under pathological or stress conditions leading to Nrf2 accumulation and nuclear translocation. Once in the nuclei, Nrf2 binds the antioxidant response elements (AREs), common promoters of endogenous protective genes, among them NAD(P)H-quinone oxidoreductase (NQO1), and glutathione-*S*-transferase (GST), as well as heme-oxygenase 1 (HO-1) and superoxide dismutase 1 (SOD-1) [96] which are studied herein, initiating the transcription and protein expression of the antioxidant genes. The studies reported herein indicate that through Nrf2 activation MTZ, MEL, and Trolox not only counterbalance the Aβ-mediated ROS generation but also the concomitant metabolic/bioenergetic changes. Reinforcing the link between Nrf2 activation and restoration of mitochondrial metabolic parameters, it has been shown that canonical Nrf2 activators as the electrophilic agent SFN as well as other natural antioxidants not only counteract Aβ-mediated apoptotic and oxidative mechanisms but also protect from amyloid-induced alterations in mitochondrial respiration and ATP production both in neuronal cultures and transgenic mouse models [97–102]. In spite of their potential for the development of future therapeutic strategies, the consequences of the prolonged use of Nrf2 activators should be further explored, specially taking into consideration the reported association of Nrf2 exacerbated activation with certain types of cancer [103].

Different mechanisms have been reported as responsible for the activation of Nrf2. Until recently, the prevailing view was that Nrf2 was primarily regulated through the Keap1 (Kelch-like ECH-associated protein 1) pathway. Keap1, as part of the E3 ubiquitin ligase complex together with Cullin 3 and Ring-Box 1, is able to interact with Nrf2 allowing its ubiquitination and subsequent proteosomal degradation, a mechanism that maintains Nrf2 low endogenous levels [104, 105]. As illustrated in Fig. 11, electrophilic molecules are capable of interfering with this Nrf2 degradation path by chemically modifying specific sensor Cys residues in the Keap1 molecule leading to conformational changes that prevent its binding to Nrf2 and its subsequent proteosomal degradation, ultimately resulting in the nuclear translocation and activation of the transcription factor [104, 106]. Although Keap1 is the most studied regulator of Nrf2 activity, more recently, the role of another E3-ubiquitin ligase adaptor, β-TrCP (β-transducing repeat-containing protein), was described [49]. This mechanism, independent of Keap1 (Fig. 11), is regulated by glycogen synthase kinase 3 (GSK-3) which phosphorylates Nrf2 targeting it for proteosomal degradation upon binding to the multi-protein complex formed by β-TrCP together with the Skp1 adaptor, Cullin 1, and Rbx1 [107, 108]. Cell signaling pathways such as PI3K/Akt, able to phosphorylate and inactivate GSK-3, inhibit Nrf2 degradation and result in its nuclear translocation and activation [49].

It is known that Nrf2 is compromised by age [109, 110] and downregulated in AD [111], deficits replicated in different animal models [112, 113]. Notably, our data indicate that Aβ challenge did not translate in a concomitant activation of Nrf2 and its downstream antioxidant response elements in spite of eliciting a severe ROS production in neuronal cells, findings consistent with the Nrf2 downregulation coexisting with the amply documented oxidative stress reported in AD [111, 114]. Along this line, it is noteworthy to mention that Aβ has been shown to activate GSK-3 signaling in vitro, a finding consistent with the increased activity of the enzyme observed in AD brains [115]. Thus, it is tempting to speculate that, based on GSK-3 role as a key element in one of the major pathways regulating Nrf2 ubiquitination and proteosomal degradation, Aβ-mediated GSK-3 activation may account for the lack of Nrf2 activation and nuclear translocation even in the presence of the exacerbated Aβ-mediated ROS generation reported herein.

The presence of MTZ, MEL, and Trolox—compounds that completely inhibited Aβ-mediated ROS generation and restored ATP production—induced the activation and nuclear translocation of Nrf2 in both SH-SY5Y and primary neurons pointing out to a crucial role of the transcription factor in the prevention of Aβ-mediated neuronal dysfunction. Whether other protective pathways are additionally targeted by the compounds remains to be investigated. Our work, through the use of different inhibitors affecting Keap-1 and β-TrCP degradation pathways, demonstrates that all three Aβ-protective compounds, in difference to the electrophilic compound SFN, activate Nrf2 and subsequent downstream antioxidant proteins through the PI3K/GSK-3 axis and not through disruption of the Nrf2-Keap-1 complex. The precise mechanisms by which MTZ, MEL, and Trolox affect PI3K/GSK-3 action remain to be elucidated. In the case of melatonin, limited reports suggest a role for melatonin receptor in the activation of the pathway [116, 117], but to our knowledge, no receptor has been described for MTZ. However, irrespective of the underlying mechanisms, the Nrf2 activation and concomitant induction of downstream antioxidative proteins likely account for the protective effect of the compounds on Aβ-mediated dysfunction.

Underscoring the pharmacologic potential of the Aβ-protective compounds employed in the current work for future prospective therapeutic strategies, it should be noted that both MTZ and MEL are FDA-approved drugs with well-studied bioavailability, organ distribution, and pharmacokinetics. As it is the case of Trolox, MTZ and MEL have been shown to be well-absorbed and cross the BBB [23, 118–121] and, as a result, have the potential to be active in vivo. Indeed, highlighting the relevance of our work and the potential of antioxidant treatment for neurodegenerative disorders, limited published work indicates that MEL administration preserved age-dependent cognitive impairment in APPPS1 transgenic mice [122] while Trolox protected this transgenic line from its characteristic neuritic alterations [123]. Further studies are needed to completely demonstrate the in vivo efficacy of MTZ, MEL, and Trolox as pharmacologic compounds targeting Aβ-mediated alterations and capable of ameliorating oxidative damage, restoring mitochondrial function and metabolic/bioenergetic abnormalities in transgenic models.

## Conclusions

Overall, the data presented herein provide insight into the detrimental effect of Aβ for mitochondrial function and the metabolic/bioenergetic changes that correlate with dysregulation of neuronal activity in AD. The work delineates the molecular mechanisms by which MTZ, MEL, and the vitamin E analog Trolox protect from Aβ-mediated detrimental alterations and concomitant metabolic changes identifying their common mechanistic activity as Nrf2 activators through the PI3K/GSK-3 axis and validating the relevance of these pathways as targets for pharmacological intervention. Based on the unsuccessful outcome of numerous AD clinical trials—among them those aimed at decreasing Aβ levels through diminishing its production or increasing its brain clearance, as well as those protecting from age-related oxidative stress—it is becoming clearer that targeting only one element of the complex interlinked cellular pathways affected by the disease will not be sufficient. Successful prevention/delay of AD development will more likely require complex strategies encompassing multiple genes and pathways. The findings reported herein suggest that modulation of the PI3K/Akt path—likely an early event in the disease process leading to both suppression of the stress response and decreased neuronal survival—may constitute attractive targets. The use of small molecule Nrf2 activators may offer additional approaches either as preventive agents or in combination therapies with other treatment options to address the detrimental effects of Aβ on mitochondrial and synaptic function as well as the metabolic/bioenergetic abnormalities encompassing the complex and multifactorial pathways leading to AD pathogenesis.

## Supplementary information

**Additional file 1: Figure S1.** Kinetic of cellular oxygen consumption in a Seahorse platform. The graph is a schematic example of real time changes in OCR values using the Cell Mito Stress Assay in a Seahorse metabolic analyzer. The figure depicts the injection time-points of the different modulators of cellular respiration and illustrates the kinetic data analysis employed by Report Generator software to evaluate the fundamental parameters of mitochondrial function: basal respiration, ATP production, proton leak, maximal respiration, spare respiratory capacity, and non-mitochondrial respiration.

**Additional file 2: Figure S2.** Quantitation of Nrf2 nuclear fluorescence induced by Methazolamide, Melatonin, and Trolox in the presence of PI3K- and GSK-3 inhibitors. Graph depicts the quantitation of Nrf2 nuclear fluorescence signal from images depicted in Figure 13. Fluorescence of at least 400 cells was quantitated utilizing ImageJ software and expressed in fold of control cells. Data is represented as mean ± SD; **** indicates *p*<0.0001.

**Additional file 3: Figure S3.** Quantitation of cytoplasmic SOD-1 and HO-1 fluorescence induced by Methazolamide, Melatonin, and Trolox in the presence of PI3K- and GSK-3 inhibitors. Graphs in Panels A and B depict the quantitation of the cytoplasmic SOD-1 and HO-1fluorescence signal, respectively, of at least 400 cells utilizing ImageJ software and expressed in fold of control. Data is represented as mean ± SD; **** indicates *p*<0.0001.

## Data Availability

The datasets used and/or analyzed during the current study are available from the corresponding authors on reasonable request.

## Abbreviations

AD: Alzheimer’s disease
Akt: Protein kinase B
Aβ: Amyloid-β
EM: Electron microscopy
FCCP: p-trifluoro methoxy-carbonyl cyanide phenylhydrazine
GSK-3: Glycogen synthase kinase 3
Keap1: Kelch-like ECH-associated protein 1
MEL: Melatonin
MTZ: Methazolamide
Nrf2: Nuclear factor erythroid-derived 2-like 2
PI3K: Phosphatidylinositol 3-kinase
RIPA: Radioimmunoprecipitation assay
ROS: Reactive oxygen species
SFN: Sulforaphane
TLX: Trolox
WB: Western blot
