# Correction to: Nrf2 activation through the PI3K/GSK-3 axisprotects neuronal cells from Aβ-mediatedoxidative and metabolic damage

**DOI:** 10.1186/s13195-020-00605-6

**Published:** 2020-03-24

**Authors:** Krystal Sotolongo, Jorge Ghiso, Agueda Rostagno

**Affiliations:** 1 Department of Pathology, New York University School of Medicine, 550 FirstAvenue, New York, NY 10016 USA; 2 Department of Psychiatry, New YorkUniversity School of Medicine, 550 First Avenue, New York, NY 10016 USA

**Correction to: Alzheimers Res Ther (2020) 12:13**

**https://doi.org/10.1186/s13195-019-0578-9**

After the publication of this article [1], we became aware that there were errors in Figs. 4 and 13.

Specifically: Figure 4: instead of displaying the appropriate images, the 1μM Aβ+Trolox panel duplicated the NoAβ+MTZ image and the 1μM Aβ+MTZ panel duplicated the 10μM Aβ+MTZ image. Both errors have been corrected.

Figure 13: the Trolox+SB216763 panel that inadvertently duplicated the Noactivator+SB216763 image has been replaced. There was also an imbalanced resizing of the NoInhibitor+MEL panel which has now been replaced for a different original image from the same experiment. The correct Figures 4 and 13 are shown below.

**Fig. 4:**
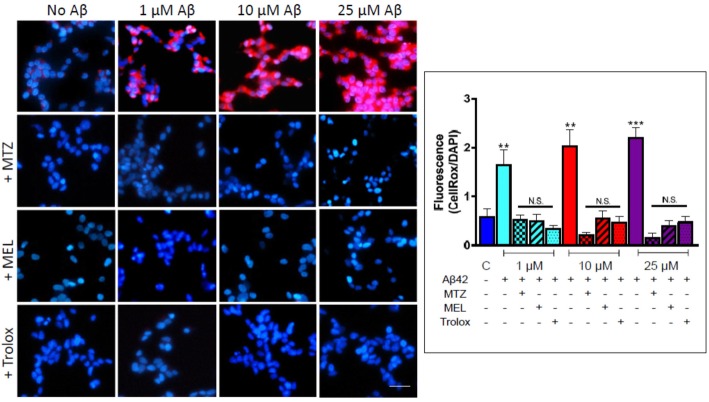
Methazolamide, melatonin, and Trolox protect from Aβ-mediated ROS generation in SH-SY5Y. Following 24 h incubation with Aβ42 (0–25 μM) in the presence or absence of MTZ (300 μM), MEL (100 μM), and Trolox (300 μM), ROS-generated species were detected with CellROX 5 μM), and nuclei counterstained with Hoechst (1 μg/ml). Images depict CellRox fluorescence (red signal) and DAPI DNA counterstaining; bar, 25 μm. The graph on the right illustrates the quantitation of CellROX fluorescence values normalized to DAPI signal using ImageJ analysis software; data is represented as mean ± SEM. **p < 0.01 and ***p < 0.001

**Fig. 13:**
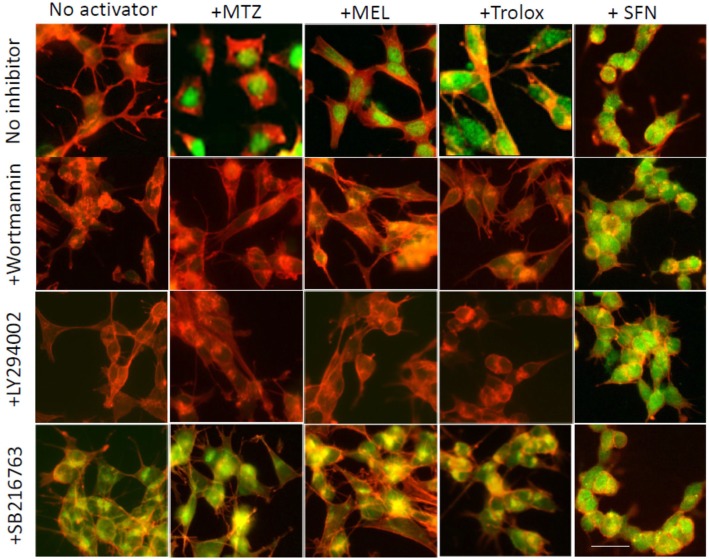
Methazolamide, melatonin, and Trolox activate Nrf2 through a PI3K-mediated pathway. SH-SY5Y cells were treated with MTZ (300 μM), MEL (100 μM), or Trolox (300 μM) in the presence of the PI3K inhibitors LY294002 and Wortmannin (10 μM each) or the GSK-3 inhibitor SB216763 (10 μM). As a control, cells were incubated with SFN (5 μM), a compound capable of activating Nrf2 through disruption of its binding to Keap-1, a PI3K-independent pathway. In all cases, Nrf2 expression was evaluated by immunocytochemistry as in Figs. 7 and 8. Green fluorescence highlights Nrf2 nuclear translocation, and red fluorescence depicts actin staining with Alexa 588-conjugated phalloidin. Bar represents 20 μm in all images. Quantitation of the nuclear fluorescence signal is shown in Additional file 2: Figure S2
