# Supplementary figures and images for: Nrf2 activation through the PI3K/GSK-3 axis protects neuronal cells from Aβ-mediated oxidative and metabolic damage
Source: Alzheimers Res Ther. 2020 Jan 13;12:13. doi: 10.1186/s13195-019-0578-9 (PMC6958642; doi:10.1186/s13195-019-0578-9)

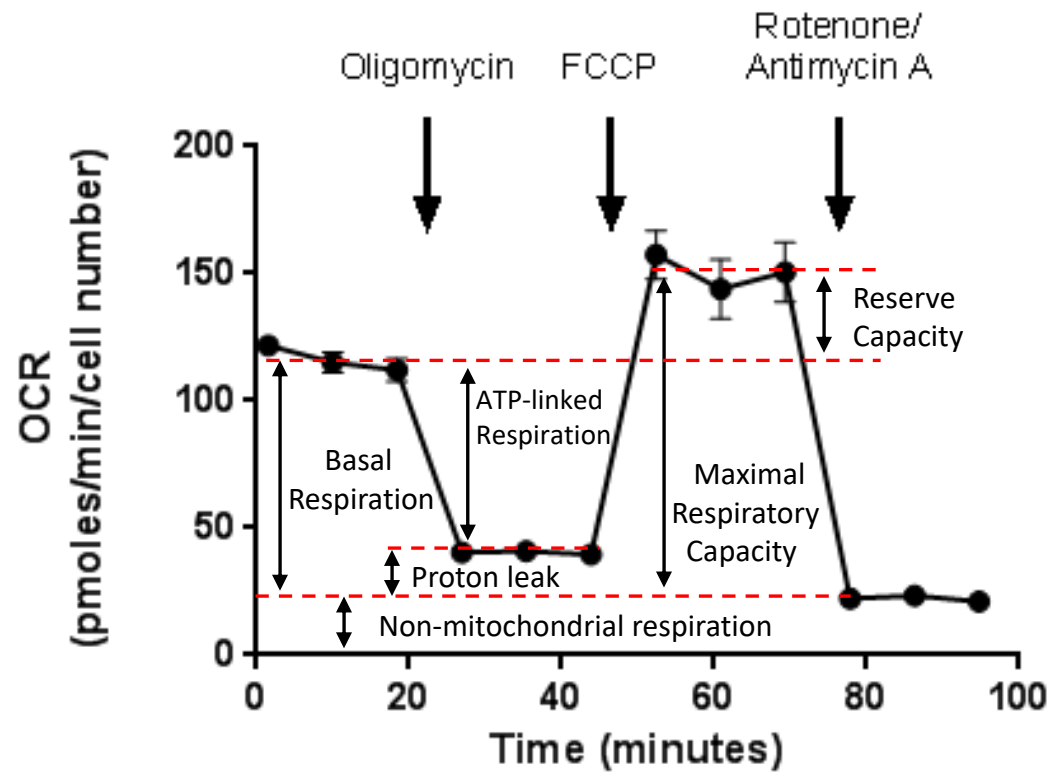

Supplemental Figure 1

Supplement: Supplementary file 1 — Additional file 1: Figure S1. Kinetic of cellular oxygen consumption in a Seahorse platform. The graph is a schematic example of real time changes in OCR values using the Cell Mito Stress Assay in a Seahorse metabolic analyzer. The figure depicts the injection time-points of the different modulators of cellular respiration and illustrates the kinetic data analysis employed by Report Generator software to evaluate the fundamental parameters of mitochondrial function: basal respiration, ATP production, proton leak, maximal respiration, spare respiratory capacity, and non-mitochondrial respiration. [file 13195_2019_578_MOESM1_ESM.pdf]

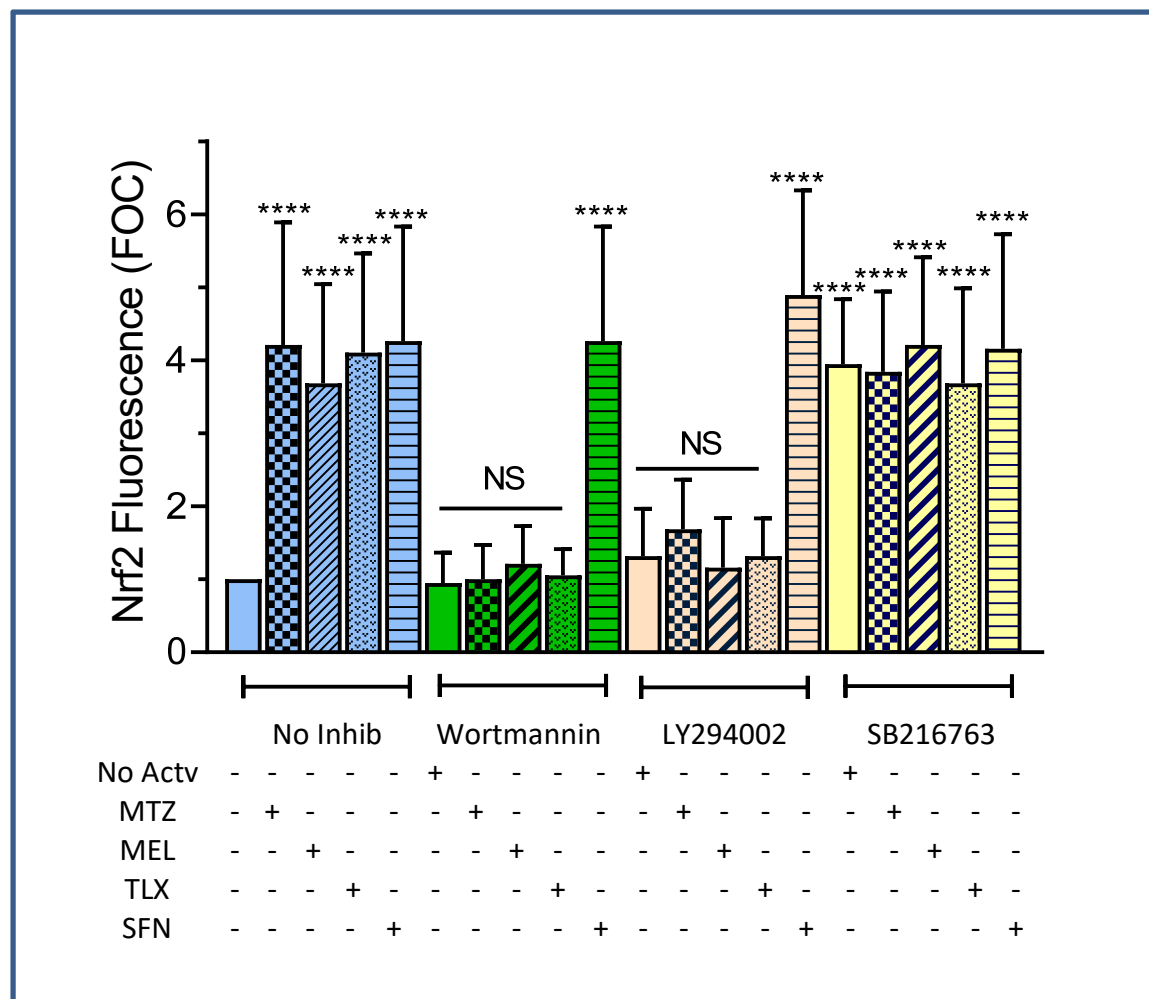

Supplementary Figure 2

Supplement: Supplementary file 2 — Additional file 2: Figure S2. Quantitation of Nrf2 nuclear fluorescence induced by Methazolamide, Melatonin, and Trolox in the presence of PI3K- and GSK-3 inhibitors. Graph depicts the quantitation of Nrf2 nuclear fluorescence signal from images depicted in Figure 13. Fluorescence of at least 400 cells was quantitated utilizing ImageJ software and expressed in fold of control cells. Data is represented as mean ± SD; **** indicates p<0.0001. [file 13195_2019_578_MOESM2_ESM.pdf]

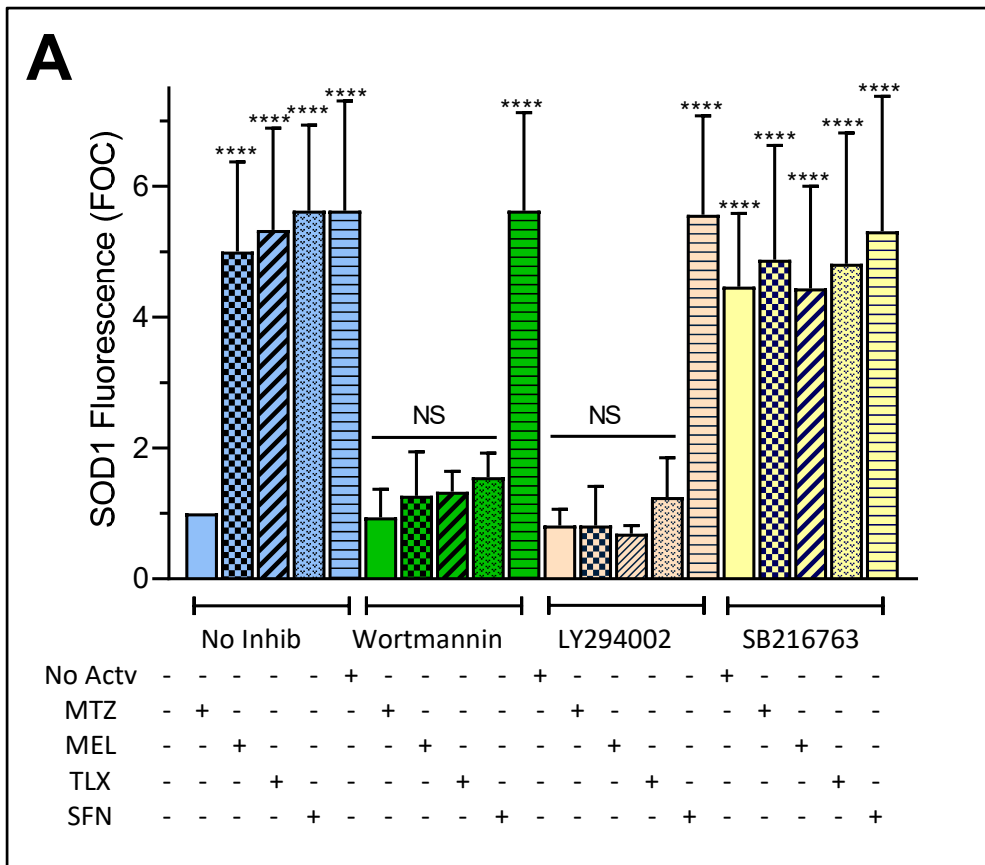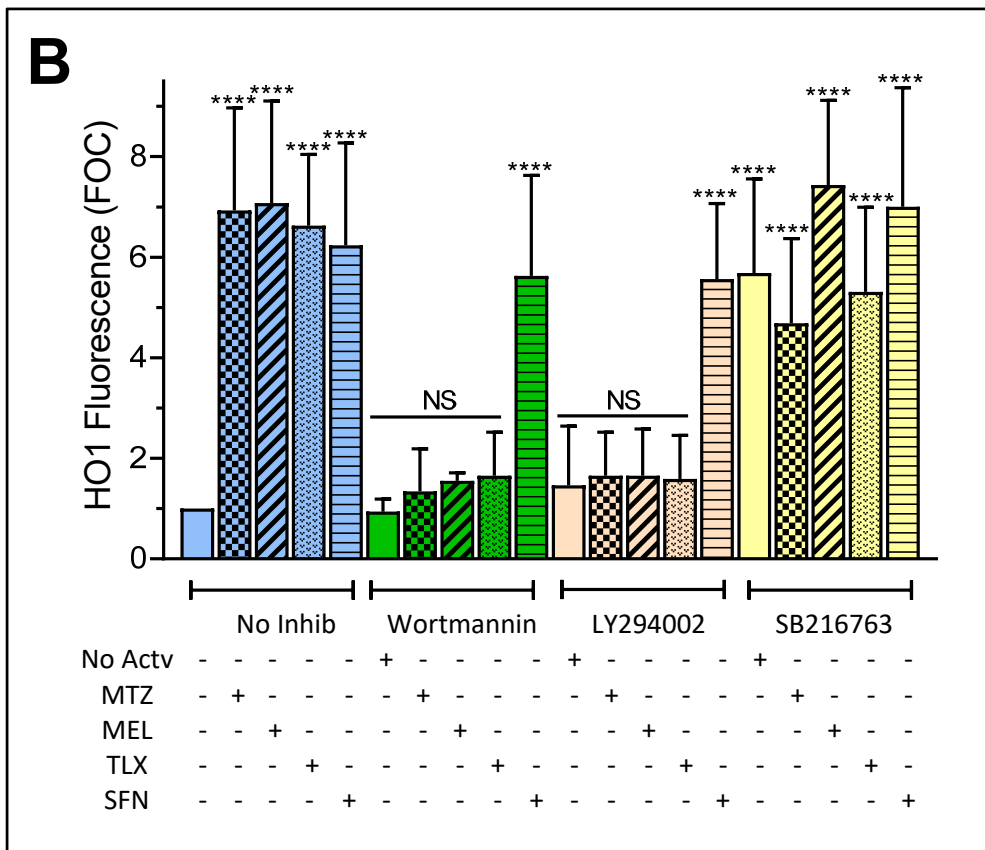

Supplementary Figure 3

Supplement: Supplementary file 3 — Additional file 3: Figure S3. Quantitation of cytoplasmic SOD-1 and HO-1 fluorescence induced by Methazolamide, Melatonin, and Trolox in the presence of PI3K- and GSK-3 inhibitors. Graphs in Panels A and B depict the quantitation of the cytoplasmic SOD-1 and HO-1fluorescence signal, respectively, of at least 400 cells utilizing ImageJ software and expressed in fold of control. Data is represented as mean ± SD; **** indicates p<0.0001. [file 13195_2019_578_MOESM3_ESM.pdf]
